# Enhanced glucose production in norepinephrine and palmitate stimulated hepatocytes following endurance training

**DOI:** 10.3389/fphys.2024.1514082

**Published:** 2024-12-17

**Authors:** Ken D. Sumida, Vera M. Lordan, Casey M. Donovan

**Affiliations:** ^1^ Department of Health Sciences, Chapman University, Orange, CA, United States; ^2^ Department of Biological Sciences, USC Dornsife, University of Southern California, Los Angeles, CA, United States

**Keywords:** exercise, liver, gluconeogenesis, lactate, norepinephrine, fatty acids

## Abstract

Enhanced hepatic gluconeogenesis plays an important role in exercise glucose homeostasis when hepatic glycogen stores are depleted. Livers from trained animals demonstrate greater rates of gluconeogenesis in the presence of elevated substrate with and without hormonal stimulation. Training has been reported to have a particularly profound impact on norepinephrine-stimulated gluconeogenesis, but this was only demonstrated in the presence of other gluconeogenic hormones. Here we reexamine the impact of endurance training on norepinephrine-stimulated gluconeogenesis in the absence of any other hormones. Isolated hepatocytes from trained and untrained rats were incubated in 6 mM lactate with various concentrations of norepinephrine (0 nM–20 nM). Absent norepinephrine, gluconeogenic rates were significantly greater from trained hepatocytes compared to controls (97.2 ± 6.7 vs 57.6 ± 8.7 nmol/mg protein; *p* < 0.01). In the presence of NE (0.5–20 nM), gluconeogenesis from trained liver cells was significantly greater at all NE concentrations compared to controls. The NE-stimulated increase in gluconeogenesis above basal (0 nM NE) was also greater for trained vs control (36% vs 19%, respectively). Concomitant with the max NE-stimulated increase in gluconeogenesis, lactate uptake was significantly elevated for trained vs. control hepatocytes (307.22 ± 44.5 vs 124.5 ± 23.9 nmol/mg protein; *p* < 0.01), with lactate uptake quantitatively accounting for the entire increase in gluconeogenesis for trained hepatocytes. Endurance training was also observed to significantly elevate glucose production in presence of 0.6 mM palmitate, both in the absence and presence of NE. These findings confirm that hepatocytes from endurance-trained animals demonstrate enhanced rates of NE-stimulated gluconeogenesis, as well as palmitate-stimulated glucose production.

## Introduction

It is well known that endurance training results in an enhanced resistance to exercise-induced hypoglycemia (Bergman et al., 2000; Brooks and Donovan, 1983; Brooks and Donovan, 1983; Donovan and Brooks, 1983; Donovan and Sumida, 1990; Holloszy and Coyle, 1984). This is generally attributed to an increase in fat oxidation resulting in a “sparing” of blood glucose and liver glycogen (Coggan et al., 1990; Coggan et al., 1995; Gollnick, 1985; Holloszy and Coyle, 1984; Fan et al., 2017). However, over the past few decades, increasing evidence has emerged in support of an additional adaptation, i.e. an increased capacity for *de novo* glucose synthesis (Bergman et al., 2000; Donovan and Brooks, 1983; Donovan and Sumida, 1997; Podolin et al., 1994a; Sumida et al., 1993; Sumida et al., 2003). Under conditions in which endurance trained animals demonstrate true resistance to exercise-induced hypoglycemia, the resistance to hypoglycemia was shown to derive from an improved hepatic glucose production via gluconeogenesis, not decreased glucose uptake (Brooks and Donovan, 1983; Donovan and Sumida, 1990). Enhanced gluconeogenesis has also been reported for trained humans when exercising at the same relative workload as untrained subjects, i.e. a greater workload at the same percent of VO_2_ max (Bergman et al., 2000).

In attempting to elucidate the mechanism(s) involved, we provided the first direct evidence that endurance training specifically increased the gluconeogenic capacity of the liver for both lactate and alanine (Sumida et al., 1993; Sumida and Donovan, 1995). While these initial observations were made in the absence of any of the hormones known to enhance glucose production during exercise, subsequent investigations by our group (Sumida et al., 2003) and others (Drouin et al., 1998; Podolin et al., 1994a; Podolin et al., 1994b) confirmed this training effect in the presence of glucagon, epinephrine and norepinephrine (NE). In general, these studies have reported training-induced increases in hormone-stimulated hepatic gluconeogenesis of 25%–46%, similar to what we reported in the absence of hormones (Sumida et al., 1993; Sumida and Donovan, 1995). The exception to these moderate elevations in training-induced hepatic gluconeogenesis was the work of Podolin et al. (1994b) who reported NE stimulated elevations in gluconeogenic rates that were over 100% for trained, while untrained animals demonstrated no significant response. This was a rather surprising observation given the general perception that NE plays a negligible role in hepatic glucose production during exercise (Camacho et al., 2005), though this has not been tested rigorously following endurance training. However, it remains unclear as to whether the magnitude of the observed difference in gluconeogenic rates for NE versus other glucoregulatory hormones reflects a true difference in training adaptations or simply reflects the different conditions under which they were studied. In contrast to other studies, Podolin et al. (1994b) employed a unique liver slice preparation that preferentially directed carbon flow to glycogen (99%), relied primarily on ^14^C incorporation from ^14^C-lactate for the measurement of gluconeogenesis, and included several other hormones known to influence gluconeogenesis (i.e. glucagon, insulin and epinephrine).

Given that NE demonstrates a relatively greater response to exercise than either glucagon or epinephrine (Galbo et al., 1977a; Galbo et al., 1977b; Kjaer et al., 1989; Winder et al., 1982) and the potential significance of these earlier findings (Podolin et al., 1994b) in explaining improved resistance to exercise-induced hypoglycemia following training, we sought to re-examine the adaptation in NE stimulated gluconeogenesis. Using primary isolated hepatocytes, we examined the dose-response for lactate uptake, net molar glucose production and ^14^C-lactate incorporation into glucose at concentrations of NE ranging from 0 to 20 nM. Further, given that this training adaptation in gluconeogenesis is most likely manifest *in vivo* during the later stages of endurance exercise when glycogen stores are depleted and free fatty acid levels elevated, we examined the impact of adding palmitate, a fatty acid known to stimulate gluconeogenesis, both in the absence and presence of NE (González-Manchón et al., 1988).

## Methods

*Animals & Training:* The experimental protocol for this study was pre-approved by the University of Southern California Institutional Animal Care and Use Committee and in accord with the Public Health Service policy on the use of experimental animals for research. Female Wistar rats, 8 weeks of age upon arrival, were housed individually in a temperature-controlled room, had free access to food and water, and were kept on a reverse 12-hour light/dark cycle. Animals were allowed 1 week to acclimate, then randomly assigned to either a control (n = 14) or endurance-trained group (n = 14). Trained animals were run on a rodent treadmill (Quinton model 42–15) 5 days/week for 10 weeks with the running time and speed progressively increased over the first 4 weeks from 20 m/min at 10% grade for 10 mins to 30 m/min at 10% grade for 90 min with the workload maintained until the end of week 10. Control animals were acclimated to treadmill running 2 days/wk, 10 min/day at 20 m/min, 10% grade for the final 4 weeks (i.e., weeks 7–10).

Forty-eight hours prior to the experiment, all animals engaged in treadmill running in order to minimize any residual effects from the last exercise bout. All animals ran at 25 m/min, 10% grade for 45 min constituting the final exercise bout for the trained animals and an acute exercise bout for the controls. 24 hours before the experiment, all food was withdrawn to deplete glycogen stores and minimize glucose production via glycogenolysis. Water continued to be provided *ad libitum* to all animals. On the day of the experiment, an equivalent number of trained and control animals were used with all solutions prepared on that day.

*Hepatocyte Preparation:* Isolation of the liver was accomplished as previously described (Sumida et al., 1993; Sumida et al., 2003; Sumida and Donovan, 1995). Specifically, after the surgical isolation of the liver, the animal was placed in a humidified and temperature controlled (37°C) plexiglass perfusion chamber, identical to the perfusion chamber described in detail by Exton and Park (Exton and Park, 1967). Before entering the liver, the perfusate was sequentially filtered through a nylon mesh, oxygenated (95%:5%, O_2_:CO_2_), and then passed through a bubble trap. Hepatocytes were isolated essentially as described by Berry and Friend (Berry et al., 1991). Briefly, the liver was perfused with a medium containing 160 mM Na^+^, 5.4 mM K^+^, 0.8 mM Mg^2+^, 139 mM Cl^−^, 0.8 mM SO_4_^2-^, 1.0 mM PO_4_^−^ and 25 mM HCO_3_^−^, at a rate of 4.5 mL g^-1^ min^-1^. The first 50 mL of perfusate were discarded following a single pass prior to establishing a recirculation mode. Collagenase (0.05%) was then added to the perfusion medium, and the liver was perfused for an additional 20 min to allow for degradation of connective tissue. During the perfusion with collagenase, the right hindlimb musculature (i.e. soleus, gastrocnemius, plantaris, vastus, gracilis and biceps femoris) was removed and frozen for subsequent analysis of citrate synthase. At the end of the perfusion, the liver was carefully removed from the animal and placed in a beaker containing 100 mL of perfusate with 1 mM CaCl_2_ added. Scissors were used to open any remaining intact liver capsules and gently stirred until there was a concentrated homogenate of cells. Cells were then filtered through two layers of a nylon mesh and centrifuged for 2 min at 40 x g. The supernatant was aspirated, and the cells washed 3 more times with the perfusate/CaCl_2_ solution. Cells were maintained on ice or kept in the refrigerated centrifuge throughout the washing procedure. Following the final wash, the cells were rapidly weighed and reconstituted (100 mg cells/mL incubation medium) in fresh incubation medium (see below). Prior to reconstituting the cells, a small aliquot (20ul) of the cell suspension was tested for viability using a Trypan blue exclusion test. Preparations demonstrating less than 90% viability were excluded from the study.

*Incubations with Norepinephrine:* Cell suspensions (0.5 mL) from trained (n = 7) and control (n = 7) livers were added to 20 mL scintillation vials containing 1 mL of the incubation medium. The incubation medium was Krebs-Henseleit buffer with TES (30 mM) at a pH of 7.4 to which substrate was added to achieve 6 mM lactate, 0.6 mM pyruvate, and U-^14^C-lactate (24,000 dpm/mL). Each vial was then gassed for 10 s with O_2_:CO_2_ (95:5), capped, and gently shaken in a 37°C water bath for 15 min to reestablish basal metabolism and ion gradients (Berry and Friend, 1969; Chan and Exton, 1978). Following pre-incubation, aliquots were taken for measurement of glycogen, ^14^C-glycogen, protein, glucose, ^14^C-glucose, lactate, and ^14^C-lactate as described below. Norepinephrine was then added to achieve a concentration of either: 0, 0.5, 1.0, 1.5, 2.0, 5.0, and 20.0 nM. Immediately following the addition of NE, each vial was re-gassed with O_2_:CO_2_ (95:5) for 10 s, capped, and incubation period initiated for an additional 30 min at 37°C in a shaking water bath. At the end of the 30 min incubation period, one aliquot was taken and added to a tube containing potassium hydroxide for the determination of glycogen. Another aliquot was added to a tube containing sodium hydroxide for the subsequent determination of protein. Perchloric acid (8%) was then added to the scintillation vial to stop the reaction, the contents transferred to test tubes, vortexed and centrifuged for 15 min at 1,500 × g. The supernatant was then collected and stored at −80°C for subsequent analyses of glucose, lactate, ^14^C-glucose specific activity and ^14^C-lactate specific activity. Each vial was analyzed in triplicate and the average was used to assess rates of glucose production and lactate removal.

*Norepinephrine + Fatty Acids:* Aliquots (0.5 mL) of hepatocytes from trained (n = 7) and control (n = 7) animals were incubated as described above in the presence albumin-bound palmitate added to achieve a final concentration of 0.6 mM in the incubation medium. NE was also added to achieve final concentrations of 0, 0.5, 1.0, 1.5, 2.0, 5.0, and 20.0 nM. After the addition of palmitate and NE, each vial was re-gassed with O_2_:CO_2_ (95:5), capped, and incubated for 30 min at 37°C in the shaking water bath. Reactions were terminated and aliquots were analyzed as indicated above.

*Chemical Analyses:* Glucose and lactate concentrations were determined enzymatically on neutralized samples as described by Raabo and Terkildsen (1960) and Hohorst (1963), respectively. ^14^C-lactate and ^14^C-glucose specific activities were determined from samples isolated via ion-exchange chromatography as described previously (Donovan and Pagliassotti, 1989). Briefly, samples were passed through two tandem columns, the first containing Dowex 50 (H^+^ form) and the second Bio-Rad AG-8 (acetate form). Glucose was collected in the initial eluant, and lactate was subsequently eluted off the 2nd column with 0.5 M formic acid. Known concentrations were run concurrently with each group of experimental samples to assess the completeness of separation. Acid eluants were neutralized, evaporated to dryness and reconstituted with distilled water. One fraction of the reconstituted sample was then assayed for glucose or lactate as described above, while the other fraction was prepared for liquid scintillation counting (Packard TriCarb 2100 TR) for the quantification of ^14^C activity. Glycogen concentrations were determined according to Dubois et al. (1956). Protein concentrations were determined via the Bradford assay (Bradford, 1976) with bovine serum albumin used as the standard. Skeletal muscle citrate synthase (EC 4.1.3.7) activity was determined as described by Srere (Srere, 1969).

*Calculation and Statistics:* Hepatic glucose production (HGP) and lactate uptake (LAup) were calculated as the difference in their respective concentrations pre- and post-incubation, i.e. they reflect production/uptake for the 30-minute incubation period. ^14^C-glucose appearance (dpm/mg protein) reflected the difference in ^14^C-glucose activity pre- and post-incubation. All values were normalized to an incubation lactate specific activity of 4,000 dpm/μmol for purposes of comparison. To account for potential differences in cell number within the incubation medium, all values were expressed per mg of protein. Dose-response curves were plotted utilizing nonlinear regression (Prism v.10, GraphPad Software Inc.). Within and between group comparisons were made utilizing a two-way ANOVA for repeated measures, with multiple comparisons utilizing the Holm-Sidak test. Single factors between groups (e.g., citrate synthase) were analyzed via Student’s t-test. The level of significance was set at *p* < 0.05 for all comparisons and all values are expressed as the mean ± SEM.

## Results

Body weights were not significantly different between trained and control animals (251 ± 8 g vs. 254 ± 5 g; *p* = 0.76) prior to experiments. Following 10 weeks of training, skeletal muscle citrate synthase activity was significantly elevated for trained versus control animals (22.2 ± 2.6 vs. 10.7 ± 1.4 μmol/min × g^-1^; *p* < 0.001), establishing the efficacy of our training regimen for inducing a well-established marker of endurance training. Neither glycogen content (µg/mL) or ^14^C-glycogen counts (dpm/mL) were observed to increase significantly relative to pre-incubation values for either group at any norepinephrine concentration (Table 1).

**TABLE 1:** Effect of norepinephrine on hepatocyte glycogen concentration & incorporation of^14^C-lactate into glycogen.

Norepi (nM)	Glycogen *(ug/mL)*		^14^C-glycogen *(dpm/mL)*	
	Control	Trained	Control	Trained
*Bkgd*	--	--	20.1 ± 1.2	19.3 ± 1.1
*Pre-Inc*	19.4 ± 0.9	22.9 ± 1.5	21.0 ± 1.0	19.3 ± 1.2
0.0	20.5 ± 2.1	23.0 ± 1.9	27.5 ± 2.2	24.7 ± 3.4
0.5	19.1 ± 1.0	21.3 ± 2.3	28.3 ± 3.8	25.8 ± 4.1
1.0	21.5 ± 1.4	21.8 ± 0.6	28.7 ± 3.5	25.5 ± 2.1
1.5	23.3 ± 3.2	21.7 ± 1.2	26.8 ± 2.0	24.7 ± 2.2
2.0	18.2 ± 1.1	19.8 ± 1.6	24.8 ± 1.7	24.6 ± 2.2
5.0	18.2 ± 1.6	23.4 ± 2.4	27.2 ± 1.9	24.6 ± 1.7
20.0	19.3 ± 1.5	21.0 ± 1.3	27.2 ± 2.0	24.4 ± 2.4

No significant differences between Control (n = 7) and Trained (n = 7).

**FIGURE 1:**
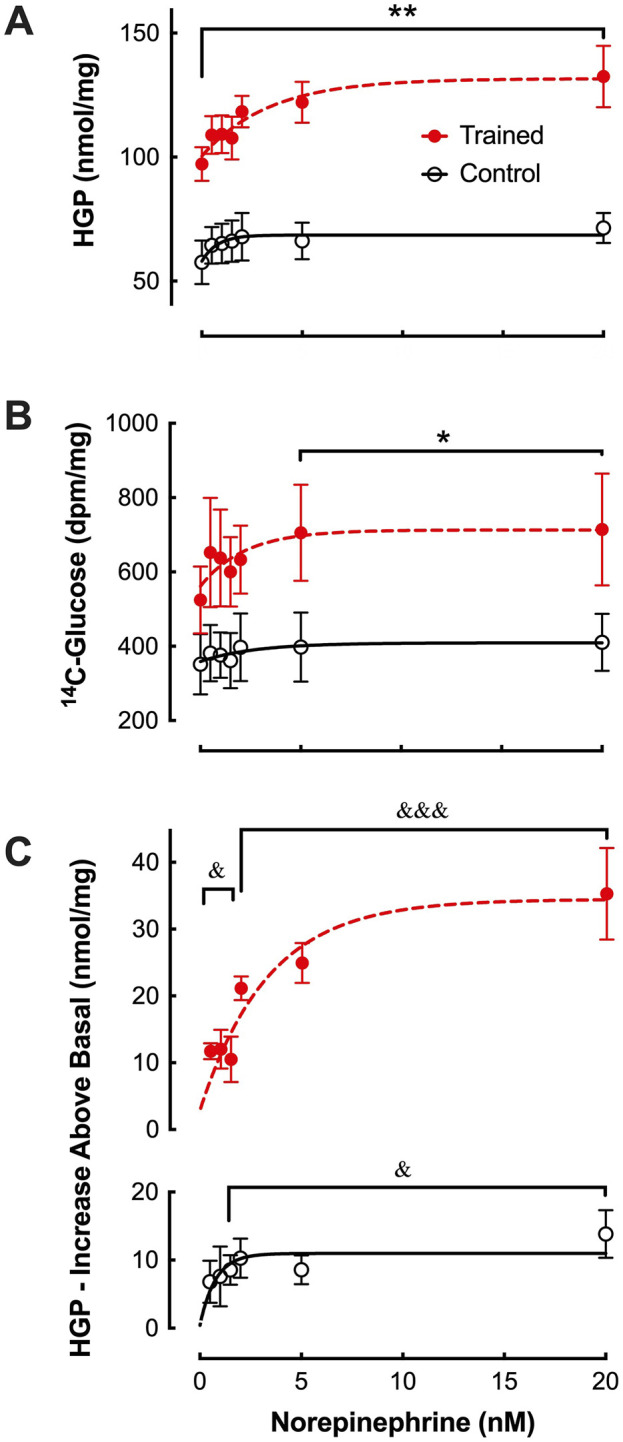
Effects of endurance training and norepinephrine concentration on **(A)** glucose production (HGP), **(B)** ^14^C-glucose production, and **(C)** glucose production above basal from control (open black circles; n = 7) and trained (closed red circles; n = 7) isolated hepatocytes. Values are expressed as means ± SEM. *p < 0.05; **p < 0.01 for control vs trained. ^&^ *p* < 0.05; ^&&&^ *p* < 0.001 for values above basal (0 nM) within group.

In the absence of NE (i.e. basal), hepatic glucose production (Figure 1) was significantly greater from the liver cells of trained animals (97.2 ± 6.8 nmol/mg) compared to controls (57.6 ± 8.7 nmol/mg; *p* ≤ 0.01). Both groups demonstrated a significant increase in NE-stimulated HGP (Figure 1C). Further, at all NE concentrations, hepatocytes from trained animals continued to demonstrate significantly greater rates of hepatic glucose production compared to controls (Figure 1A). The absolute gluconeogenic rate in response to maximal NE was approximately 85% higher from trained hepatocytes when compared to controls (132.5 ± 12.4 vs. 71.4 ± 6.1 nmol/mg; *p* < 0.01). In addition, the relative increase from basal (i.e. 0.0 mM NE) was also greater from trained liver cells at maximal NE concentration (Figure 1C), i.e. 36% increase (*p* < 0.001), compared to controls at a 19% increase (*p* < 0.05).

**FIGURE 2:**
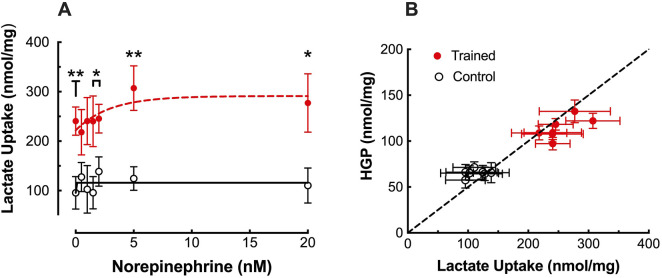
**(A)** Effects of endurance training and norepinephrine concentration on lactate uptake in control (open black circles; n = 7) and trained (closed red circles; n = 7) isolated hepatocytes. Values are expressed as means ± SEM. *p < 0.05; **p < 0.01 for control vs trained. **(B)** Hepatocyte glucose output (HGP) plotted against lactate uptake for control (open black circles; n = 7) and trained (closed red circles; n = 7) at each norepinephrine concentration. The dashed line represents the line of identity for molar equivalent glucose output versus lactate uptake.

Consistent with the increase in HGP, ^14^C-lactate incorporation into glucose was significantly elevated in hepatocytes from trained livers reaching maximal values of 714 ± 150 dpm/mg protein versus 410 ± 77 dpm/mg protein for CON (Figure 1B; *p* < 0.05). The increased ^14^C incorporation was supported by significant elevations in lactate uptake observed between hepatocytes from trained vs control animals, demonstrating a peak increase of over 140% at elevated NE concentrations (Figure 2A; *p* < 0.01). Plotting HGP against lactate uptake demonstrates that the increased lactate uptake observed in trained animals can quantitatively account for the increase in NE-stimulated hepatic glucose production as measured by cold glucose production (Figure 2B).

**FIGURE 3:**
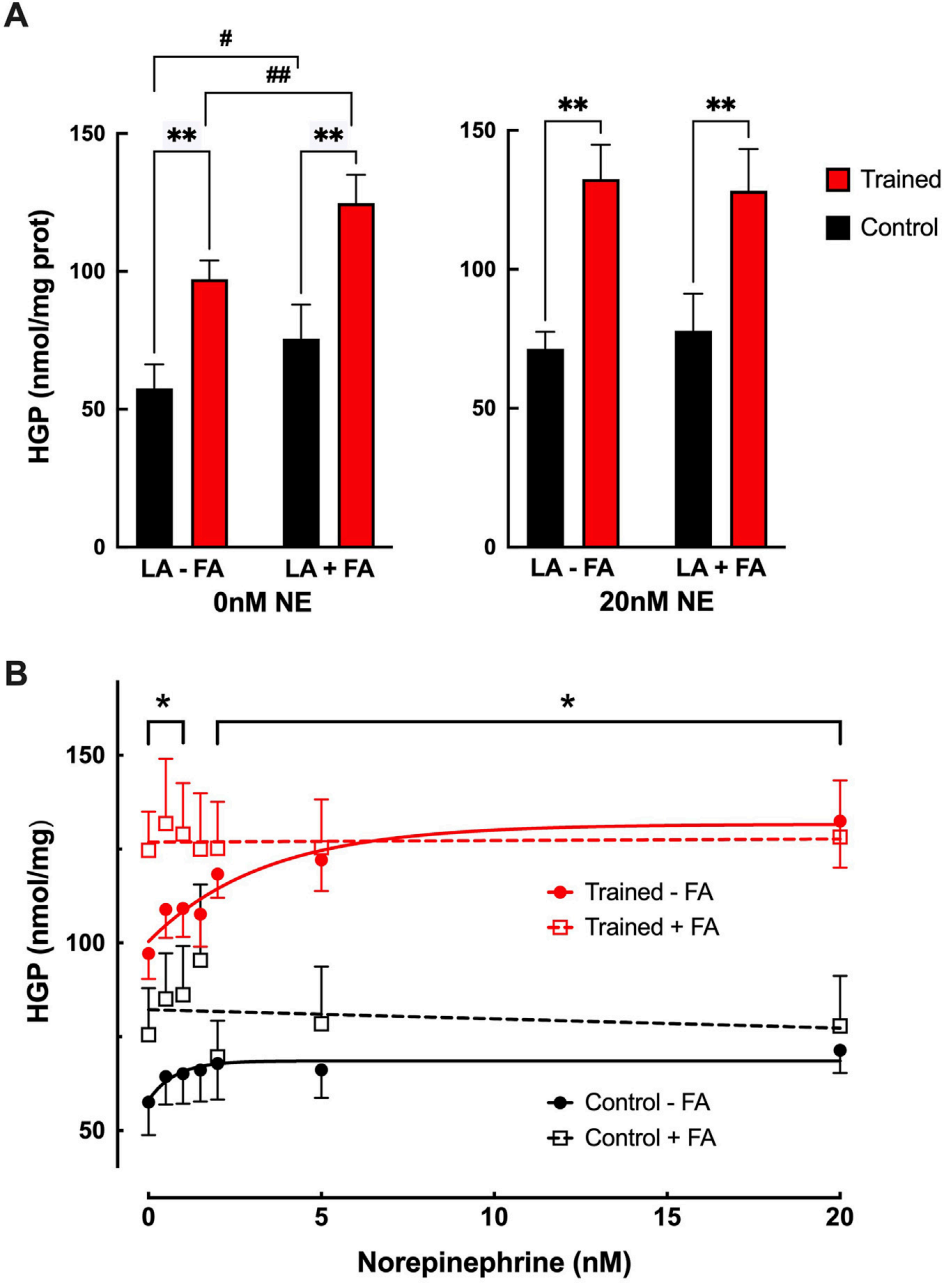
**(A)** Effects of endurance training on hepatic glucose production (HGP) for control (black bars; n = 7) and trained (red bars; n = 7) isolated hepatocytes at 0 nM or 20 nM norepinephrine (NE) in the presence of 6 mM lactate with (LA + FA) or without (LA-FA) 0.6 mM fatty acid (palmitate). Values are expressed as means ± SEM. **p < 0.01 for control vs trained. ^#^ *p* < 0.05; ^##^ *p* < 0.01 for -FA vs +FA. **(B)** Effects of endurance training and norepinephrine concentration on glucose production for control hepatocytes minus palmitate (closed black circles; n = 7), control hepatocytes plus palmitate (open black squares; n = 7), trained hepatocytes minus palmitate (closed red circles; n = 7), and trained hepatocytes plus palmitate (open red squares; n = 7). Values are expressed as means ± SEM. *p < 0.05 for control plus palmitate vs trained plus palmitate.

Adding 0.6 mM palmitate to the incubation media in the absence of NE, resulted in significantly greater glucose production (HGP) from 6 mM lactate in both control and trained hepatocytes, 75.6 ± 12.4 nmol/g vs 124.7 ± 10.3 nmol/mg, (Figure 3A; *p* < 0.05). This increase in HGP with 0.6 mM palmitate was equivalent to the maximal stimulation seen with NE for both groups (Figure 3A; *p* > 0.64) Increasing the NE concentration in the presence of palmitate failed to induce any further increase in HGP beyond basal (lactate + palmitate only) conditions (Figure 3B), yielding values similar to those obtained with maximal NE stimulation alone. While HGP was elevated for both groups in the presence of palmitate, trained hepatocytes maintained significantly greater rates of glucose production at all but one NE concentration (*p* < 0.05).

## Discussion

Consistent with our prior studies (Sumida et al., 1993; Sumida and Donovan, 1995), basal hepatic gluconeogenic rates from lactate, absent NE, were significantly greater in trained hepatocytes when compared to control cells (Figure 1). That glucose production reflected gluconeogenesis was supported by the significant depletion of hepatic glycogen following a 24 h fast with no evidence of significant glycogenolysis from any hepatocytes during the 30-minute incubation period (Table 1). Further, the significantly elevated lactate uptake reported for the trained group compared to controls, quantitatively supports the enhanced glucose production (Figure 2), as did the enhanced incorporation of ^14^C-lactate into glucose following endurance training (Figure 1B). As we and others have reported for epinephrine (Kmieć and Myśliwski, 1983; Sumida et al., 2003), hepatic glucose production was significantly elevated in hepatocytes from trained animals with NE stimulation that was sustained across all NE concentrations (Figure 1C). The higher NE-stimulated HGP for trained hepatocytes occurred in the absence of any significant glycogenolysis, confirming the gluconeogenic origin of the molar glucose released. Consistent with the observed training enhanced HGP, both ^14^C-glucose production from ^14^C-lactate and molar lactate uptake were significantly elevated following endurance training. The maximal NE-stimulated glucose production was 86% greater for liver cells from trained vs. control animals at the highest NE concentration (Figures 1, 3). Further, the hormone-stimulated increase in gluconeogenesis above basal (i.e. above 0 nM NE) was significantly greater at all NE concentrations in trained hepatocytes, while controls demonstrated a significant HGP response only at NE concentrations above 2 nM. Thus, hepatocytes from trained animals not only demonstrate greater NE stimulated rates of gluconeogenesis, but enhanced sensitivity to NE responding to lower concentrations than liver cells from control animals (i.e. 0.5 nM vs. 2.0 nM, respectively). While hepatocytes from both control and trained livers demonstrated significantly higher glucose production rates in the presence of 0.6 mM palmitate, hepatocytes from trained animals yielded rates that were 65% greater than those for controls (*p* < 0.05). Interestingly, 0.6 mM palmitate elevated glucose production rates to levels comparable with maximal NE stimulation and the addition of NE in the presence of palmitate failed to further increase HGP at any concentration of NE (Figure 3B).

The 86% increase in NE stimulated gluconeogenesis for hepatocytes from trained animals reported in the current study, is similar in magnitude to the combined glyconeogenic and gluconeogenic rates reported previously by Podolin et al. (1994b) following endurance training. Consistent with that study, we also report no significant NE stimulation of gluconeogenesis in untrained hepatocytes at norepinephrine levels below 2 nM, while hepatocytes from trained animals demonstrated significant elevations even at the lowest NE levels tested. Despite these similarities in findings between the two studies, there was one significant difference in the observed training adaptations. Podolin et al. (1994b) observed no training differences for liver gluconeogenesis in the absence of NE and thus the ∼100% increase they observed was due entirely to training enhanced NE stimulated increases. We on the other hand observed a substantial increase in gluconeogenesis for trained hepatocytes (i.e. ∼40 nmol/mg) without hormonal stimulation, something we have reported in several previous studies (Sumida et al., 1993; Sumida and Donovan, 1995; Sumida et al., 2003). This training enhanced gluconeogenic response to elevated substrate concentration alone could account for approximately 65% of the difference in HGP we observed under maximal NE stimulation. While this observed difference remains unexplained, our incubation medium at 0 mM NE contained no other hormones while that of Podolin et al. (Podolin et al., 1994b) contained several additional hormones known to impact gluconeogenesis, i.e. insulin (10^−7^ M), epinephrine (0.25 ng/mL) and glucagon (10^−7^ M). It should be noted that the added 36% increase in gluconeogenic output from trained hepatocytes observed with maximal NE stimulation in the current study is similar to that reported previously for glucagon (Drouin et al., 2004) and epinephrine (Sumida et al., 2003).

Despite increased interest in exercise training induced adaptations in the liver over the past 2 decades, this has been focused primarily on fatty acid metabolism and has provided little additional information that might explain the training enhanced increase in gluconeogenesis either in the absence or presence of NE stimulation. Regulation of gluconeogenesis primarily occurs at three levels, Glucose-6-Phosphatase (G6Pase), Fructose-1,6-Bisphosphatase (F1,6BPase) and pyruvate carboxylase/PEPCK. We have previously suggested that the level for this training adaptation(s) in gluconeogenesis most likely lies below the level of the triose phosphates (Donovan and Sumida, 1997), based on the observation that gluconeogenesis from dihydroxyacetone is not enhanced with training, despite generating higher gluconeogenic rates than either lactate or alanine (Sumida and Donovan, 1995). In support of this, neither G6Pase nor F1,6BPase activity (Takahashi et al., 2024) have been shown to increase with endurance training. Further, no training adaptations have been reported for hepatic F-2,6-BP levels during *in vitro* studies of hepatic gluconeogenesis (Horn et al., 1997). While this would seem to suggest adaptations at the level of the phosphoenolpyruvate formation, neither pyruvate carboxylase or PEPCK activity, mitochondrial or cytosolic, have been observed to increase with training (Takahashi et al., 2024). Further, while training enhances gluconeogenesis from lactate and alanine, it did not improve gluconeogenic rates from glutamine, a substrate generating comparable gluconeogenic rates (Sumida and Donovan, 1995). That the training adaptation might lie at the conversion of lactate and alanine to pyruvate is not supported by any observed increase in ALT activity (Sumida and Donovan, 1995) and while hepatic LDH activity is reduced with training, it is not accompanied by a change in the isozyme profile (Sumida et al., 1995). However, there is at least one report indicating a 30% increase in the hepatic lactate transporter, MCT2, following endurance training in mice (Lezi et al., 2013). That transport of lactate may be a critical component of training enhanced gluconeogenesis is supported by the current finding that the increase in lactate uptake could account for the entire increase in glucose output by trained hepatocytes (Figure 2B). Thus, the critical adaptations for enhance gluconeogenesis with training may lie at the level of substrate transport into the hepatocyte.

Our data would appear to indirectly support a common pathway for NE and fats in the activation of HGP from lactate as previously suggested (Taylor et al., 1983). Increasing NE concentration for hepatocytes in the absence of palmitate led to an increase in gluconeogenesis, but that rate never exceeded the glucose production of hepatocytes exposed to 0.6 mM palmitate in the absence of NE (Figure 3B). Although the precise mechanism for fatty acid stimulation of hepatic gluconeogenesis is unknown, it has been attributed to: 1) the allosteric activation of pyruvate carboxylase as a result of the increase in acetyl-CoA via beta oxidation, 2) the elevation in mitochondrial NADH that facilitates the formation of glyceraldehyde 3-phosphate, and 3) more ATP availability (Lam et al., 2003). In like manner, NE has been postulated to increase HGP via an elevation in mitochondrial redox due to the oxidation of intracellular fatty acids resulting in an increased flux through the Krebs cycle (Taylor et al., 1983). Norepinephrine has also been proposed to inhibit acetyl-CoA carboxylase resulting in an elevation in citrate and a concomitant decline in phosphofructokinase activity (Richards and Uyeda, 1982). Some caution is warranted in drawing a common mechanism for NE and fatty acid stimulation of glucose production based on our current findings, as we did not utilize ^14^C-lactate to establish the gluconeogenic origin of the glucose production (though given the low glycogen values in fasted livers, it is very likely the glucose production was gluconeogenic in origin). Further, we did not fully examine the impact of varying palmitate concentration on hepatic glucose production in trained and untrained livers.

In the current study we employed female rats, as have many other studies, owing to their willingness to run on the treadmill under an imposed load while adjusting their food consumption to maintain bodyweights comparable to sedentary controls (Applegate et al., 1982). So as to match training loads and duration for all animals in this study we did not attempt to match the estrous phase. Attempting to match estrous phase in female rats undergoing treadmill running can compromise the match in training load/duration given the known impact of such training on estrous cycles (Carlberg and Fregly, 1985; Chatterton et al., 1995), as well as the estrogen levels in trained animals (Caston et al., 1995). While we acknowledge the impact of estrogen on hepatic glucose metabolism (Kraemer et al., 2012; Yan et al., 2019) and the possible limitations that may be imposed, there are several reasons we believe this did not seriously compromise our findings. The procedures we employed for isolating and stabilizing these primary hepatocytes result in hormone responsive cells with minimal residual bound ligands thus minimizing any potential acute impact of estrogen (Berry et al., 1991). Further, both control and trained animals were exposed to an identical/final bout of exercise 48 h prior to the experiment, minimizing any acute exercise effect. Finally, both control and trained animals were housed individually minimizing any chance that the two groups would establish distinct and separate estrous cycles, i.e. cycling would be randomized within groups. It should also be noted that in prior studies of endurance training enhanced gluconeogenesis, we have employed both female (Sumida et al., 1993; Sumida and Donovan, 1995) and male rats (Sumida et al., 2003) obtaining virtually identical results without matching the estrous phase for female animals. That there is little difference between female and male rats with respect to this adaptation in hepatic gluconeogenesis is further confirmed by others employing male rats with similar results (Drouin et al., 1998; Podolin et al., 1994b). Of note, the impact of endurance training on norepinephrine-stimulated gluconeogenesis in the current study was essentially the same as that of Podolin et al. (1994b) who employed male rats.

In summary, the present results indicate that endurance training augments gluconeogenic rates in hepatocytes in the presence of elevated lactate levels, rates that are greatly augmented in the presence of norepinephrine. Specifically, hepatocytes from trained animals demonstrated an 86% increase in the maximal NE-stimulated HGP when compared to controls that was quantitatively supported by the elevation in lactate uptake and increase in ^14^C-glucose production from ^14^C-Lactate. Further, trained hepatocytes appear to demonstrate greater sensitivity to NE stimulation, responding with increased gluconeogenic rates at all concentrations of NE, 0.5–20 nM, while hepatocytes from untrained animals failed to significantly respond to any NE concentration below 2.0 nM. Finally, hepatocytes from trained animals maintained the elevation in hepatic glucose production from lactate compared to controls when incubated with palmitate both in the presence and absence of NE. For both trained and untrained hepatocytes, 0.6 mM palmitate led to an elevation in glucose production rates that were equal to those observed with maximal NE stimulation, and the addition of NE in the presence of palmitate did not lead to any further increase in glucose output. Collectively, these findings support a robust adaptation to exercise training that supports enhanced gluconeogenesis in the presence of elevated lactate and is enhanced by both norepinephrine and fatty acid stimulation.

## Data Availability

The original contributions presented in the study are included in the article/supplementary material, further inquiries can be directed to the corresponding author.
